# Treatment outcomes using CBT-IA with Internet-addicted patients

**DOI:** 10.1556/JBA.2.2013.4.3

**Published:** 2013-12-13

**Authors:** Kimberly S. Young

**Keywords:** Internet use disorder, Internet addiction, treatment outcomes, cognitive-behavior therapy

## Abstract

*Background and aims:* Internet Gaming Disorder, a subtype of Internet Addiction, is now classified in Section 3 of the *DSM-5*. Cognitive behavioral therapy (CBT) has been suggested in treating Internet addiction as this modality has been shown to be an effective treatment for similar impulse control disorders. Given the daily and necessary use of the Internet and technology in general compared to other compulsive syndromes, a specialized form of CBT has been developed called Cognitive-Behavioral Therapy for Internet Addiction (CBT-IA). CBT-IA is a comprehensive three phase approach that includes behavior modification to control compulsive Internet use, cognitive restructuring to identify, challenge, and modify cognitive distortions that lead to addictive use, and harm reduction techniques to address and treat co-morbid issues associated with the disorder. *Methods:* As the first model of its kind, this study examines 128 clients to measure treatment outcomes using CBT-IA. Clients were evaluated using the Internet Addiction Test (IAT) to classify subjects and were administered twelve weekly sessions of CBT-IA. Treatment outcomes were measured at the end of the twelve weeks, one-month, three months and at six month post-treatment. *Results:* Results showed that over 95% of clients were able to manage symptoms at the end of the twelve weeks and 78% sustained recovery six months following treatment. *Discussion and Conclusions*: Results found that CBT-IA was effective at ameliorating symptoms associated with Internet addiction after twelve weekly sessions and consistently over one-month, three months, and six months after therapy. Further research implications such as investigating long-term outcome effects of the model with larger client populations and treatment differences among the subtypes of Internet addiction or with other cultural populations using CBT-IA are discussed.

## INTRODUCTION

Early studies on Internet addiction originated in the United States (e.g.,Young, 1996) and soon a rapidly growing number of countries such as Italy (Ferraro, Caci, D’Amico & Di Blasi, 2007), Pakistan (Suhail & Bargees, 2006), and the Czech Republic (Simkova & Cincera, 2004) found similar problems due to Internet addiction. China has struggled with similar issues and in 2007 the country restricted game use to less than three hours a day (Jaffe & Uhls, 2011) and the Korean government has built a network of 140 Internet-addiction counseling centers, in addition to treatment programs at almost 100 hospitals and the Internet Rescue camp, a forested area about an hour south of Seoul to treat the most severe cases. The camps are entirely paid for by the government, making it tuition-free (Sang-Hun, 2010).

Today, Internet Use Disorder is included in Section 3 for further research in the upcoming *DSM-5,* the bible of American psychiatric medicine. Researchers have likened Internet addiction to impulse-control disorders on Axis I in *DSM-IV* (e.g., Aboujaoude, Koran, Gamel, Large & Serpe, 2006; Beard & Wolf, 2001; Block, 2008; Shapira et al., 2003; Young, 1998) and have used various forms of *DSM-IV* based criteria to define Internet addiction. Cognitive-Behavior Therapy (CBT) has also been suggested as an effective treatment in Internet addiction (e.g., Greenfield, 1999; Hansen, 2002), given the compulsive nature of the disorder. Given the daily and necessary use of the Internet and technology compared to other compulsive syndromes, a specialized kind of CBT was developed to treat this disorder (Young, 2011) called Cognitive-Behavioral Therapy for

Internet Addiction (CBT-IA), a uniquely designed model for treating Internet addicts applying CBT with harm reduction therapy (HRT). This study is the first to measure treatment outcomes using CBT-IA to treat serious cases of Internet addiction.

## WHAT IS CBT-IA?

CBT in general allows addicts to understand addictive feelings and actions while learning new coping skills and ways to prevent a relapse. CBT usually requires three months of treatment or approximately twelve weekly sessions. With Internet addicts, it has been suggested that the early stage of therapy should be behavioral, focusing on specific behaviors and situations where the impulse control disorder causes the greatest difficulty (Hall & Parsons, 2001). As therapy progresses, the focus is more on the cognitive assumptions and distortions that have developed and the effects of these on behavior (Young, 2007). Maladaptive cognitions such as overgeneralizing or catastrophizing, negative core beliefs, and cognitive distortions also contribute to compulsive use of the Internet (Caplan, 2002; Davis, 2001; LaRose, Mastro & Eastin, 2001). In one of the first outcome studies for internet addiction, Young (2007) hypothesized that those who suffer from negative core beliefs are most drawn to the anonymous interactive capabilities of the Internet in order to overcome these perceived inadequacies.

In treating Internet addiction, abstinence recovery models are not practical as computers have become such a salient part of our daily lives. Clinicians have generally agreed that moderated and controlled use of the Internet is most appropriate to treat the problem (Young, 2010). Given the dynamics involved with Internet addiction, CBT-IA was developed to address the unique features of the problem (Young, 2011). In the first phase of the CBT-IA, behavior therapy is used to examine both computer behavior and non-computer behavior. Computer behavior deals with actual online usage, with a primary goal of abstinence from problematic applications, while retaining controlled use of the computer for legitimate purposes (Young, 2011).

Internet addicts feel a sense of displacement when online and were unable to manage central aspects of their lives due to their growing preoccupation with online use (Young, 2004). They start to miss important deadlines at work, spend less time with their family, and slowly withdraw from their normal routines. They neglect social connections with their friends, coworkers, and with their communities, and, ultimately, their lives become unmanageable because of the Internet. As the addiction grows, they become consumed with their Internet activities, preferring online games, chatting with online friends, or gambling over the Internet, and ignoring family and friends in exchange for solitary time in front of the computer (Leung, 2007). Managing their time online and offline is an initial goal of CBT-IA (Young, 2011).

In the second phase, cognitive therapy is used to address denial that is often present among Internet addicts and to combat the rationalizations that justify excessive online use.

Initially, therapy addresses the maladaptive cognitions that serve as triggers that initiate binge-behavior over the Internet. For instance, some Internet addicts suffer from distorted thoughts about the self that include rumination (e.g., constantly thinking and worrying about the problems associated with the individual’s online use) and extreme self-concepts favoring the online self (e.g., “I am worthless offline, but in the online world I am someone”). They may also suffer distorted thoughts about the world such as “Nobody loves me” and “The online world is the only place that I am respected”. These exaggerated thoughts are characterized by all or nothing thinking that can intensify and perpetuate the patient’s Internet addiction. For example, if a gamer creates and controls an avatar (an online game character) who can achieve various goals in online games, he or she may perceive the offline real world as less desirable, which leads to his or their psychological dependence on using the Internet to improve or maintain his or their self-esteem. Internet addicts may also develop a cognitive bias that they are better treated by others in the virtual world and feel discomfort or dissatisfaction with their real lives.

CBT-IA uses cognitive restructuring to break this pattern. Cognitive restructuring helps put the client’s thoughts “under the microscope” by challenging him or her and re-scripting the negative thinking that lies behind him or her. In doing so, CBT-IA can help clients understand that they are using the Internet to avoid situations or feelings. Our moods are driven by what we tell ourselves, and this is usually based on our interpretations of our environment. Cognitive restructuring will help clients re-evaluate how rational and valid these interpretations are. For instance, a client who uses online games as a way to build self-esteem will start to see that they are using the Internet to satisfy needs that are not being fulfilled in his or her real life.

Once clients become aware of their patterns of faulty thinking, they can begin to challenge these thoughts more independently of therapy. In this way, they will find it more difficult to rationalize or justify their Internet use and to break the cycle of associating Internet use with a better life. To help the client stay focused on moderated treatment goals, CBT-IA helps clients identify the major problems or consequences caused by addiction to the Internet.

The third phase of CBT-IA uses Harm Reduction Therapy (HRT; Marlatt, Blume & Parks, 2001) for continued recovery and relapse prevention. As situational factors play a role in the development of Internet addiction, HRT can be used to identify and treat psychiatric issues co-existing with compulsive Internet use and treat social issues in immediate family and/or marital relationships. HRT addresses any co-existing factors associated with the development of Internet addiction. These factors can include personal, situational, social, psychiatric, or occupational issues. Often, addicts falsely assume that just stopping the behavior is enough to say, “I am recovered”. Full recovery is more than simply refraining from the Internet. Complete recovery means investigating the underlying issues that led up to the compulsive behavior and resolving those them issues in a healthy manner; otherwise, relapse is likely to occur.

CBT-IA is a new and untested therapy. As Internet addiction is a growing concern, this study examines client treatment outcomes using this model. While many researchers have suggested treatment approaches to address Internet addiction, little has been studied on actual therapy outcomes. This study employed a survey research design to assess the utility and application of CBT-IA. This study utilized validated measures to classify Internet addicts from non-addicts and utilized standardized tools to measure problematic online use and problems caused by compulsive use. Outcomes measures assessed treatment goals at the end of twelve weekly therapy sessions and at one month, three months, and six months following treatment.

## METHODS

### Participants

Participants were 128 clients seen through the Center for Internet Addiction. Clients who met five or more of the criteria were included in this study (*N* = 116) and 12 clients who met four of the criteria but exhibited serious problems due to Internet use were included. Clients who exhibited high-risk behaviors such as histories of psychological trauma, sexual abuse, or Axis II pathology were excluded and sent for referrals. Candidates were screened using the Internet Addiction Test (IAT; Young, 1998). The IAT is a worldwide accepted and validated testing instrument that examines symptoms of Internet addiction such as a user’s preoccupation with Internet use, ability to control online use, extent of hiding or lying about online use, and continued online use despite consequences of the behavior.

The 20-item IAT has been validated in various countries including the USA (Widyanto, Griffiths & Brunsden, 2011), France (Khazaal et al., 2008), Germany (Brand et al., 2011), Norway (Johansson & Götestam, 2004), Finland (Kaltiala-Heino, Lintonen & Rimpelä, 2004; Korkeila, Kaarlas, Jääskeläinen, Vahlberg & Taiminen, 2010), Italy (Ferraro et al., 2007), Greece (Siomos, Dafouli, Braimiotis, Mouzas & Angelopoulos, 2008), Iran (Ghassemzadeh, Shahraray & Moradi, 2008), and China (Lam, Peng, Mai & Jing, 2009).

### Procedures

The Center for Internet Addiction was established in 1995 and their web site at www.netaddiction.com provides education, support, and treatment to people concerned about Internet addiction. Clients can seek treatment through traditional outpatient services. Adult clients requesting treatment were screened for Internet addiction using the IAT. Those classified for inclusion in the study completed an intake counseling form administered during the initial session that evaluated information related to compulsive use of the Internet for this study.

Sessions were conducted between the client and the principle investigator. Initial sessions gathered familial background, symptoms of the presenting problem, its onset, and severity. CBT-IA addressed presenting symptoms related to computer use, specifically abstinence from problematic online applications and strategies to control use. CBT-IA also focused on cognitive issues and harm reduction for underlying factors contributing to Internet abuse such as martial discord, job burnout, problems with coworkers, or academic troubles, depending upon the unique situation of each client. Use of the Internet was routinely evaluated and treatment outcomes were evaluated after 12 sessions and at one-month, three-month and six-month follow-up.

## MATERIALS

The Internet Addiction Diagnostic Questionnaire (IADQ; Young, 1998) was used to measure treatment outcomes. This 8-item questionnaire is widely used in the literature for diagnosis of Internet addiction. Meeting five or more of the criteria classifies one as addicted. The measure, given its standardization, can also be used as an outcome measure. It examines one’s preoccupation with Internet use, one’s ability to control Internet use, one’s habit to conceal or lie about his or her Internet use, one’s reliance on the Internet as a form of psychological escape, one’s interest in outside activities beyond the Internet, one’s quality of relationships, and one’s withdrawal when going without the Internet (e.g., feelings of depression or irritability).

Based on the variables identified in the literature an outcome checklist was also constructed for this study that includes ten behaviors associated with Internet addiction. The 10-item checklist was given to family or friends of the client to gain objective data of treatment successes following counseling. Questions were rated along a four-point Likert scale (1 = Never; 2 = Seldom; 3 = Sometimes; 4 = Frequently) to force clients on a narrow choice of answers. Questions assessed counseling effectiveness on the following targeted treatment goals:

1. The client sticks to a structured schedule of Internet use and doesn’t eclipse the targeted number of total hours online each week set as a therapy goal.
2. The client’s spouse, parent, or other loved one tells him or her that they have seen a difference in their Internet habits and the client’s behavior toward them.
3. The client keeps a strict accounting of the money spent for online activities (say for access to virtual casinos or gaming sites) and stays within a budget.
4. The client performs work tasks in a timely fashion that closely resembles their former pattern before turning to the Internet.
5. The client rediscovers those favorite hobbies and activities they used to enjoy.
6. The client expends greater energy communicating with those in their daily lives than to strangers on the Internet.
7. The client sees others obsessed with Internet in a different light, with an understanding that they are creating problems for themselves and those closest to them.
8. When the client does use the Internet for legitimate reasons or for limited recreational purposes, he or she feels less and less tempted to resume old habits.
9. The client feels a greater desire to go out with a spouse or a family member and socialize with friends, turning down fewer invitations and making more of your own.
10. The client looks back at his or her time of addiction to see a different person from a different period of time.

## Instrument validation

To determine the validity and reliability of the outcome checklist, mental health practitioners in the CBT therapy field evaluated the instrument as well as through a pilot test. Three therapists whom practiced some form of CBT therapy were asked to evaluate the content of the instrument and to comment on the clarity and appropriateness of the items. Before implementing the survey, a pilot test was also administered to five randomly selected college students to check the time required to finish the questionnaire, to determine if there were ambiguity and format problems, and clarity of questionnaire items. Only minor adjustments were made in the checklist accordingly. Data were analyzed and percentages with frequencies were calculated for the dichotomous items and content analyses were used to evaluate qualitative data.

## Ethics

The study procedures were carried out in accordance with the Declaration of Helsinki. The Institutional Review Board of the Center for Internet Addiction approved the study. All subjects were informed about the study and all provided informed consent.

## RESULTS

A total of 128 clients were evaluated. Demographically, 35% of clients were women and 65% of clients were men. Mean age for clients ranged from 22 to 56. The 63% were Caucasian, 2% were African-American, and 25% were of Asian decent, 23% held a Master’s degree or doctorate, 62% held a four-year Bachelor’s degree, and 5% had earned a high school diploma.

Table 1 outlines the problematic applications reported by clients based on gender. Men were most addicted to online pornography (39%), online gaming (31%), online gambling (14%), sexual online chat rooms (13%), and miscellaneous activities such as social media (2%), respectively. Women were most addicted to online chat rooms, both sexual (16%) and general (16%), online shopping (29%), online auction houses (9%), online gaming (7%), and miscellaneous activities such as social media (24%). Overall, patients were addicted to role-playing games and pornography as primary activities with online shopping, social media, and chatting (including instant messaging or dating sites) all being a close third of online problems.

**Table 1.** Problematic online applications by gender

Online activity	Females	Males	Total
Chat (Sexual)	16% (7)	13% (11)	14% (18)
Chat (General)	16% (7)	0% (0)	5% (7)
Pornography	0% (0)	39% (32)	25% (32)
Gambling	0% (0)	14% (12)	9% (12)
Gaming	7% (3)	31% (26)	23% (29)
Auction houses	9% (4)	0% (0)	3% (4)
Shopping	29% (13)	0% (0)	10% (13)
Other (social media, etc.)	24% (11)	2% (2)	10% (13)
Total	35% (45)	65% (83)	100% (128)

Table 2 presents results from the IADQ at the onset of treatment. Results showed that 116 subjects met five or more of the eight-item criteria contained in the IADQ, 12 met four of the criteria and also exhibited serious problems due to Internet overuse. Specific problems were reported: creating harmful consequences due to use (94%), psychological escape associated with use (92%), excessive use (90%), preoccupation with Internet use (83%), poor time management (87%), and concealment of use (87%) were the most frequently reported behavioral problems. Withdrawal (57%) and loss of control (56%) where the least frequently reported problems.

**Table 2:** IADQ responses at onset of treatment

Criteria	‘YES’ responses	‘NO’ responses	Missing data
Preoccupation	83% (106)	17% (22)	0
Poor time mgt.	87% (109)	13% (17)	2
Loss of control	56% (72)	44% (56)	0
Withdrawal	57% (73)	43% (55)	0
Loss of interests	90% (115)	10% (13)	0
Created harm	94% (120)	6% (8)	0
Concealed use	87% (109)	13% (17)	2
Escape	92% (118)	8% (10)	0

Table 3 examines means and standard deviations across the eight variables of the IADQ. The IADQ was re-administered after twelve weekly therapy sessions of CBT-IA then again after one month, three months, and six months following treatment. Using Paired sample *t*-tests to test whether the changes are significant or not against the whole population at each time point is compared. At twelve weeks, patients reported marked improvement on their ability to control their use of the Internet, lying or hiding their Internet use from others, and withdrawal signs associated with reduced or eliminated Internet use. After twelve sessions, clients felt that they were able to refrain from Internet use during the weekly sessions as they provided additional support. They no longer hid their use, although that was part of treatment for them to also tell others of their problem and involve family members into their therapies. Clients also had to time to prepare themselves for life without the Internet. After twelve weeks, clients felt less preoccupied with using the Internet and showed moderate improvement in stopping to use the Internet as a form of psychological escape. Relationship problems were moderately improved after twelve weeks of therapy, however these problems gradually declined the longer the client was away from treatment. After reviewing one month to six months differences, there is significant agreement that the behaviors the client exhibited at discharge from treatment, or after the twelve sessions, clients now focused on their own time management of the computer independent of therapy. After six months, clients reported that the longer they were away from the Internet the more preoccupied that they became on using it again. Clients also reported that not being able to use the Internet as an escape was difficult the longer that they were away from treatment as they were forced to confront those issues that they neglected due to their addiction to the Internet. This could previous marital problems or family issues, or difficulties with social anxiety and making new relationships offline. The largest problem was resolving relationship problems. Six months following treatment, clients reported that relationship problems were hard to resolve, even after therapy, as some of the problems resulted from long-standing issues in their relationships. The Internet was their vice in fending off real-life difficulties so their need to go back online increased over time. Regaining interest in other activities beyond the Internet was the hardest behavior to overcome. Clients reported that the Internet had consumed most of their lives, and finding activities outside of their online use was difficult. While they were able to change their behavior in the months following treatment, they had little motivation to form new goals or interests because they did not feel the same enjoyment in those activities as they did about their Internet use. It kept them preoccupied with returning to their online activities and suggests that strong emotional bonds toward the Internet are formed among those addicted.

**Table 3.** Comparisons of the means (SD) of IADQ at the fives measurement points

	T_0_ (Before treatment)	T_1_ (After the 12th session)	T_2_ (One month after treatment)	T_3_ (Three months after treatment)	T_4_ (Six months after treatment)	*t*-test T_0_–T_1_	*t*-test T_1_–T_2_	*t*-test T_2_–T_3_	*t*-test T_3_–T_4_
Preoccupation	5.00 (0.55)	3.01 (0.08)	4.28 (0.91)	4.58 (0.16)	4.06 (0.30)	3.45**	3.20**	3.59**	3.89**
Poor time mgt.	4.40 (0.20)	1.06 (0.01)	1.12 (0.51)	1.36 (0.52)	1.90 (0.91)	2.75*	2.50*	2.40*	2.75*
Loss of control	4.30 (0.24)	1.14 (0.16)	1.66 (0.51)	1.67 (0.42)	1.76 (0.08)	3.01**	2.47**	3.20**	2.90**
Withdrawal	5.00 (0.68)	1.09 (0.88)	1.45 (0.09)	1.66 (0.78)	1.98 (0.09)	1.98**	1.75**	2.45**	2.56*
Loss of interests	4.80 (1.06)	4.67 (0.87)	3.45 (0.98)	3.04 (0.09)	2.65 (0.08)	4.12**	4.25**	4.55**	4.84**
Created harm	4.95 (0.65)	3.09 (1.09)	3.78 (0.97)	4.39 (0.84)	4.29 (0.84)	4.24**	4.13**	4.25**	4.54**
Concealed use	4.20 (0.23)	1.35 (0.12)	2.05 (0.06)	1.58 (0.52)	2.09 (0.41)	2.10*	2.30*	2.21*	2.45*
Escape	4.40 (0.63)	3.89 (0.98)	4.98 (0.78)	4.67 (0.64)	4.98 (076)	4.57**	4.69**	5.20**	5.80**
Total IADQ	4.63 (0.53)	2.41 (0.52)	2.84 (0.60)	2.86 (0.49)	2.96 (0.43)	3.28*	3.16*	3.48*	3.72**

* *p* < 0.05, ** *p* < 0.01.

**Table 4.** Comparisons of the means (SD) of outcome checklist ratings at the fives measurement points

	T_0_ (Before treatment)	T_1_ (After the 12th session)	T_2_ (One month after treatment)	T_3_ (Three months after treatment)	T_4_ (Six months after treatment)	*t*-test T_0_–T_1_	*t*-test T_1_–T_2_	*t*-test T_2_–T_3_	*t*-test T_3_–T_4_
Maintains structure	0.80 (0.17)	1.45 (0.24)	1.28 (0.31)	1.58 (0.46)	1.56 (0.33)	1.89*	1.22*	1.56*	1.65 *
Perception of others	0.38 (0.91)	2.56 (0.71)	2.32 (0.76)	2.36 (0.23)	2.90 (0.91)	2.03**	2.14**	2.59**	2.87 **
Money spent online	0.02 (0.03)	1.35 (0.31)	1.25 (0.36)	1.45 (0.33)	1.46 (0.41)	2.44**	2.55**	2.15**	2.43 **
Performs chores	0.60 (0.35)	1.95 (0.51)	0.85 (0.50)	1.25 (0.52)	1.10 (0.61)	3.96**	3.88**	3.68**	3.98 **
Rekindles interests	0.50 (0.90)	4.32 (0.35)	4.25 (0.76)	4.05 (0.52)	4.67 (0.91)	4.12*	4.25*	4.28*	4.54 *
Communicates better	0.30 (0.09)	4.35 (0.10)	4.28 (0.10)	4.05 (0.37)	4.40 (0.67)	2.14**	2.45**	1.88**	2.99 **
See other addicts	0.78 (0.12)	3.10 (0.11)	3.25 (0.26)	3.35 (0.22)	3.10 (0.25)	3.46**	2.68**	3.25**	3.54 **
differently Limits use	0.12 (0.67)	3.95 (0.31)	4.25 (0.36)	4.38 (0.32)	4.15 (0.31)	4.10**	4.12**	4.56**	4.99 **
Socializes with others	0.86 (0.90)	3.35 0.31	4.15 (0.33)	4.35 (0.54)	4.11 (0.41)	5.98**	5.15**	5.72**	5.87 **
Sees online use	0.29 (0.48)	1.05 0.40	1.35 (0.36)	1.45 (0.32)	1.32 (0.31)	1.98**	1.69**	1.95**	2.01 **
differently Total	0.58 (0.58)	3.42 (0.42)	3.40 (0.51)	3.03 (0.48)	3.60 (0.64)	4.01**	3.77**	3.95**	4.35**

* *p* < 0.05, ** *p* < 0.01.

Table 4 shows the *t*-test results from the Outcome Checklist after twelve weekly sessions, one month, three months, and six months post-treatment. The focus was to see if relapse occurred after termination of therapy. Notably, the majority of clients reported relief from the ten outcomes after twelve weeks, which suggested CBT-IA was effective and the majority of clients maintained a healthy recovered state upon six months follow-up. Maintaining a structured schedule improved significantly following twelve weekly sessions. A large part of CBT-IA is behavior modification and structured use of the Internet for legitimate use, so this is relatively consistent with the goals of therapy. The behavior was sustained at repeated checks over six months following treatment. As the results suggest, clients were able to sustain structured use of the Internet, one of the most important variables of success (Young, 2011). The loved one’s perception of the client improved as the client showed consistent recovery. The money spent on online activities was stopped or significantly reduced after twelve weeks of treatment and this remained consistent as long as the client was in recovery. The ability to perform work chores improved as clients had more time to focus on work and chores around the house without interruption from excessive use of the Internet. Consistent with the IADQ findings, the ability to rekindle hobbies and interests was the most difficult to achieve even months after the end of therapy. Client showed slight improvement even over time following treatment. The ability to communicate with others improved the longer they were away from treatment and in recovery. After six months post-treatment, clients reported that with continued practice of their therapy goals to refrain from inappropriate or unneeded use of the Internet, they were forced to develop new skills in talking with others. Through the development of new hobbies, things that took them outside of the house, they slowly were able to develop new relationships through face-to-face communication. The longer they were away from the computer the more they were able to see others who use the Internet in a different light. They were able to differentiate healthy from unhealthy or excessive use of the Internet. In serious causes, they were able to identify excessive use of the Internet resulting in problems as obsessive and compulsive use, putting into light their own previous use. This variable was highly significant to maintaining their recovery. If they could continue to view excessive Internet use as unhealthy the more likely they were to remain committed to their own sobriety from the online application they found problematic or compulsive. Initially, while in treatment, data also show that clients were able to limit ability to limit online use to legitimate reasons because it was monitored and clients were therapeutically accountable for what they did online and times they used technology. However, their ability to control or limit Internet use became more difficult the longer that they were away from therapy. In some cases, clients stated that Internet use was difficult to avoid in legitimate purposes such as checking stock information, online shopping, or making hotel reservations. Results also suggest that clients were able to be more social with others the longer they avoided the Internet. Again, consistent with the IADQ, clients reported that when they were forced to make relationships in face-to-face environments at work or school, it gradually became easier to socialize with others. While the results show that clients were able to limit Internet use and develop new social relationships over time following therapy, many noted that they still had relationships problems such as social anxiety that were not extinguished solely by limiting giving up their Internet use. Finally, results showed the clients were able view Internet addiction differently over time. Clients reported that the longer they reduced or moderated their own Internet use, they more they saw how addictive the behavior had become, and the more they viewed others addictive or compulsive use as unhealthy that served to hurt the quality of their relationships and well-being.

Overall, results suggest that the majority of patients showed improvement after twelve weekly sessions of CBT-IA and overall improved symptom maintenance upon six-month follow-up. Specifically, patients were able to maintain motivation to quit abusing the Internet and improve online time management most effectively after CBT-IA. Many patients noted that they were able to incorporate Internet use time structure, limit use to legitimate reasons to use the Internet, and abstain from problem applications early on in treatment. More complex issues such as re-kindling hobbies and interests, improving personal perception, communicating better with others, and engaging in healthy off-line activities improved by the twelfth session. Notably, the improvement seen immediately following twelve sessions of CBT-IA were maintained and remained stable across assessment periods of one month, three months, and six months following treatment termination. Overall, patients found that the presenting symptoms of Internet addiction as measured by IADQ were remedied through CBT-IA and outcomes examining signs of healthy Internet use post-treatment were able to be maintained once counseling was completed.

## DISCUSSIONS AND CONCLUSIONS

A total of 128 clients were evaluated on the Internet Addiction Test (IAT) to assess the efficacy of CBT-IA, a uniquely designed model to treat Internet addiction. Demographically, clients tended to be male, Caucasian, and had completed at least a four-year college degree. Clients were administered twelve weekly sessions of CBT-IA. Diagnostic measures were used to assess outcomes at the end of the twelve weeks, one month, three months, and at six months at the end of treatment. Results showed that an overwhelming majority of clients were able to manage symptoms of Internet addiction as measured on the IADQ and the outcome checklist.

Most were able to fully manage their symptoms by the twelfth session. As measured by the IADQ, clients reported that CBT-IA was effective at ameliorating the common symptoms of online addiction: preoccupation with Internet use, the inability to control Internet use, the need to conceal extent of Internet use, using the Internet as a form of psychological escape, ignoring outside activities beyond the Internet, hurting relationships, and withdrawal signs. Administering the IADQ upon one-month, three-month, and six-month follow-ups also showed the over 70% of Internet addicts using CBT-IA maintained symptom management and continued recovery.

Results also showed the clients were able to achieve and maintain recovery along several behavioral indicators used in the outcome checklist. These items included signs of healthy Internet use such as clients sticking to structured schedules of Internet use, loved ones reporting a reduction in the client’s Internet use, a client improves in other facets of his or her life such as work, school, and relationships. A client regains interest in lost hobbies or interests or starts new hobbies and interests that do not involve the computer. A client is limits time online to legitimate purposes and feels less tempted to resume old habits and can look back on the addiction in a different light. Results showed improvement across these categories for the over 95% of Internet addicts after twelve weekly sessions and over 78% maintained symptom management and continued recovery upon one-month, three-month, and six-month follow-up after therapy. Factors in relapse included family or marital disturbance or lack of adequate IA sponsorship.

While this study provides the first empirical data to examine the efficacy of CBT-IA, further research should continue to investigate long-term treatment outcome effects of the model with larger client populations. Future research should also explore systematic comparisons with other treatment modalities such as psychodynamic therapies, gestalt, group counseling, or in vivo counseling within an online community to determine their therapeutic impact and efficacy. Studies should also investigate treatment differences among the various types of Internet such as Internet gambling, online gaming, and Internet pornography addictions to see if treatment differences exist using CBT-IA along the various subtypes. As Internet Gaming Disorder enters the *DSM-5,* more research will focus on assessment and treatment of the disorder. Outcome data such as this will assist therapists in developing empirically-based treatment plans that best meet the standard of care for clients. It is important to have outcome data as new treatment centers emerge and the profession needs to build appropriate treatment for this new client population.
